# Mobile Mindfulness Meditation for Cancer-Related Anxiety and Neuropathy: Protocol for a Randomized Controlled Trial

**DOI:** 10.2196/47745

**Published:** 2024-02-12

**Authors:** Ariel Siritorn Orasud, Mai Uchiyama, Ian Pagano, Erin Bantum

**Affiliations:** 1 Cancer Prevention in the Pacific University of Hawai’i Cancer Center Honolulu, HI United States

**Keywords:** cancer, cancer survivorship, mindfulness meditation, mHealth, well-being, quality of life, mobile health, coaching, coach, mindfulness, meditation, survivor, survivorship, oncology, mental health, anxiety, neuropathy, survivors, RCT, randomized, clinical trial, clinical trials

## Abstract

**Background:**

Anxiety- and cancer-related neuropathy are two persistent effects related to treatment for cancer. Mindfulness meditation has been used with substantial impact as a nonpharmacologic intervention to mitigate side and late effects of treatment. Mobile apps are ubiquitous for most of the general population, yet have a particular relevance for cancer survivors, given that physical and geographic limitations can be present.

**Objective:**

This study aims to describe an ongoing trial of the Mindfulness Coach mobile app for cancer survivors.

**Methods:**

In this randomized waitlist controlled trial, cancer survivors experiencing anxiety- or cancer-related neuropathy (200 for neuropathy and 200 for anxiety) and who had finished primary cancer treatment were invited to participate. Data were collected at 3 time points regardless of randomization condition: baseline, 8 weeks, and 16 weeks. Both face-to-face and web-based recruitment strategies were used. The trial was opened for 2 separate primary outcomes (anxiety- or cancer-related neuropathy). The goal was not to compare these groups but to compare treatment and waitlist groups for each condition. In addition to evaluating the impact of mobile mindfulness on reported anxiety- or cancer-related neuropathy, other pain, fatigue, trauma, sleep, and satisfaction with the Mindfulness Coach app will also be assessed.

**Results:**

Outcomes of the study are expected in early 2024.

**Conclusions:**

Mindfulness meditation has become widely practiced, and the use of mobile technology has become ubiquitous. Finding ways to deliver mindfulness meditation to people who have been treated for cancer allows for the intervention to be accessible to a larger number of survivors. The results of this intervention could have implications for further understanding the impact of mindfulness meditation on 2 persistent side and late effects of treatment of cancer, namely anxiety- and cancer-related neuropathy.

**Trial Registration:**

ClinicalTrials.gov NCT03581357; https://ClinicalTrials.gov/study/NCT03581357

**International Registered Report Identifier (IRRID):**

DERR1-10.2196/47745

## Introduction

### Background

The latest report from the American Cancer Society estimates that there are 16.9 million cancer survivors in the United States [1]. A diagnosis of cancer or recurrence of cancer can cause psychological distress, including symptoms of anxiety and depression [2]. For patients receiving treatment for cancer, 10.3% meet the clinical criteria for an anxiety disorder [3], compared with 7% of those in the general population [4]. The prevalence of anxiety disorders is reported to be even higher for those who have been living with cancer for more than 2 years, with 17.9% of survivors meeting diagnostic criteria for anxiety [5]. As cancer treatments have become more effective than in previous decades, there are more cancer survivors living longer with cancer [6], yet also living with side and late effects of treatment.

Anxiety can manifest in both physical and psychological symptoms that negatively impact quality of life and are associated with a substantially increased risk of cancer incidence and cancer-specific mortality [7]. Psychological distress can affect a number of cancer outcomes, including quality of life, adherence to treatment, health behaviors, and potentially disease progression and survival, as well as increased use of health care resources [2].

Neuropathy is another common symptom of either cancer treatment or, less frequently, cancer itself and can be acute or chronic in nature [6]. There are a wide range of ways in which patients describe this experience, although peripheral neuropathy is often described as the sensation of shooting or stabbing pain and tingling or loss of feeling in the affected areas. This can impact many activities, such as walking, balance, and mood [6]. In patients receiving neurotoxic chemotherapy, such as platinum drugs, taxanes, vinca alkaloids, proteasome inhibitors, and immunomodulatory drugs, approximately 30%-40% of patients will develop chemotherapy-induced peripheral neuropathy (CIPN) [8]. CIPN is a common cause of pain and decreased quality of life for patients with cancer undergoing treatment as well as cancer survivors [9]. In approximately 50% of people who experience CIPN, this condition will become chronic [10].

### Mindfulness and Mindfulness Meditation

Historically conceptualized in Buddhist spiritual practice with the purpose of cultivating compassion and alleviating suffering, mindfulness meditation has been practiced for thousands of years [11,12]. Mindfulness can be described as bringing nonjudgmental attention or awareness to one’s moment-to-moment experience [12]. Mindfulness meditation is the setting aside of time to specifically engage in what is often a seated meditation practice where participants are instructed to close their eyes and are guided through a breathing and focused attention process [13].

The practice of mindfulness with the goal of improving well-being has been described as a universal human capacity that does not involve a particular cultural or religious belief system [11,12]. In recent decades, mindfulness has gained popularity in the United States and has been found to have numerous psychological and physical benefits, with a growing body of research on clinical applications of mindfulness to treat various conditions [2,11,14-20]. Mindfulness has been studied as a therapy to reduce symptoms of depression and anxiety [2], improve outcomes for chronic pain [14], reduce stress and improve attention [15], improve brain function and connectivity [16], and improve immune function [17].

The practice of mindfulness has been shown to improve quality of life and be related to more positive mental health in cancer survivors [18]. Recent studies indicate that mindfulness-based interventions have a positive impact on depression, anxiety, and quality of life in patients with ovarian cancer [2], men living with cancer [19], and patients with metastatic non–small cell lung cancer and their caregivers [20], to name just a few.

### Web-Based Behavioral Therapy for Anxiety- and Cancer-Related Neuropathy

While mindfulness is gaining a larger evidence base for applications in the clinical setting, lack of trained personnel, time constraints, reimbursement issues, and patient and provider availability are barriers to delivery and participation in a traditional, face-to-face mindfulness course [21]. Researchers in a recent face-to-face mindfulness-based stress reduction (MBSR) intervention for young adult cancer survivors identified barriers to participation reported by eligible patients who declined to participate in the intervention [22]. Out of the 446 eligible people who declined to participate, the majority reported distance to the intervention site as the main factor (41.5%), followed by time constraints (21.7%). A subset of individuals who declined were asked if they would participate in the course if it were offered in a different location or format. Of those asked, 73.8% stated that they would enroll in an MBSR course offered closer to their home, and 70.8% stated that they would enroll in a online MBSR course. Of the individuals who stated interest in a online MBSR course, 70.9% stated that they would enroll in a self-directed course delivered through educational modules [22]. These barriers to face-to-face mindfulness interventions limit access to care and therefore make face-to-face interventions less likely to be a realistic treatment model [21], but online interventions for mindfulness provide a potential avenue to overcome these barriers and increase accessibility to effective mindfulness interventions.

Advances in technology have allowed for the delivery of effective interventions in the patient’s home through a mobile app [23]. When used to effectively disseminate evidence-based interventions, apps have the potential to increase access to evidence-based health care and the use of evidence-based practices by the general population [21,23]. Previous studies have shown that mindfulness-based interventions via the web involving guided meditation audios are feasible and acceptable for cancer survivors [24], who may be too ill or lack the resources to travel to a face-to-face mindfulness intervention. A recent study using the Calm mobile app for mindfulness meditation found that the use of the app for 10 minutes daily for 4 weeks was substantially effective in reducing symptoms of depression and anxiety in patients with myeloproliferative neoplasm but did not substantially impact sleep disturbance [25].

The current American Society of Clinical Oncology clinical practice guideline does not recommend any pharmacologic agents in preventing CIPN, and there is limited evidence for the treatment of existing CIPN with pharmacologic agents, with a moderate recommendation for the use of duloxetine [26]. Previous studies suggest that online interventions may have the potential to improve conditions involving chronic pain. A study involving an online, self-guided cognitive- and behaviorally-based pain management intervention for cancer survivors experiencing CIPN had substantially greater improvements in “worst pain” compared with individuals receiving usual care [27].

Mindfulness Coach, an iOS- and Android-based mobile app, is designed to deliver a mobile mindfulness training course designed by researchers from the US Department of Veterans Affairs [21,28]. The senior author of this paper, ER, was part of the team that developed the most recent version of the app. Initially developed among other mobile apps in collaboration with the US Department of Defense [28], Mindfulness Coach is freely available to the general public for both iOS and Android operating systems. A recent study exploring the effects of the app among the general population found that use of the Mindfulness Coach app was feasible, and increased use was strongly correlated with an increase in scores measuring mindfulness characteristics including observing, describing, acting with awareness, nonreactivity to inner experience, and nonjudging of inner experience, as measured by the Five-Facet Mindfulness Questionnaire Short Form (FFMQ-SF) [21,29]. Although mobile health is increasingly being used in research and clinical settings, a recent systematic review and meta-analysis concluded that there is still inconclusive evidence due to small nonsubstantial effects of interventions, as well as concerns regarding study quality [30].

Given the persistent effects of diagnosis and treatment and the limitations of in-person offerings in most places, the Mobile Mindfulness Study was conducted to test the impact on anxiety- and cancer-related neuropathy after 8 weeks of use of the Mindfulness Coach mobile app. The study contains the following secondary aims: evaluate the impact of mobile mindfulness on general pain, fatigue, trauma, and sleep, in addition to evaluating the satisfaction with the Mindfulness Coach mobile app. The goal of this paper is to describe the study design.

## Methods

### Study Design

The study is being conducted with a randomized waitlist control design, such that participants were randomized through Qualtrics to either begin the intervention immediately or after 8 weeks. In addition, participants were invited to participate with the goal of impacting anxiety- or cancer-related neuropathy as a primary outcome (additional discussion on this below). The goal was to have 200 people complete the 8-week survey measures in each group. Funding was originally garnered to examine anxiety as a primary outcome, with additional seed funding received through the University of Hawai’i Cancer Center to test the same design for cancer-related neuropathy. These groups will not be compared with each other, rather, each treatment group (anxiety- and cancer-related neuropathy, respectively) will be compared with each waitlist control group (anxiety- and cancer-related neuropathy).

### Procedure

#### Recruitment

Recruitment closed for the study in July 2023. The University of Hawai’i (UH) Cancer Center is recognized by the National Cancer Institute as one of their designated cancer centers. The Hawai’i Cancer Consortium, to which all major medical centers on Oahu belong, is led by the UH Cancer Center. Recruitment efforts took place in oncology clinics, via the web, and through organized community events [31]. These included local medical centers and clinical offices, public events (eg, senior fair), and cancer organizations, such as the American Cancer Society and Stupid Cancer, via social media and newsletter unpaid advertisements.

#### Participants

Cancer survivors experiencing anxiety- or cancer-related neuropathy who had finished primary treatment for cancer were invited to participate in the study. They were first screened and then consented via Qualtrics, where all data were collected, as well. Inclusion criteria were the same for both groups, aside from the different scales used to measure levels of anxiety or neuropathy. Inclusion criteria for both groups include a previous diagnosis of cancer, other than nonmelanoma skin cancer; completion of primary treatment for cancer; not currently practicing meditation for more than 1 hour per week; age older than 21 years; comfortable reading and writing in English; routine access to the internet; and own a smartphone or tablet. There are no exclusion criteria.

For the anxiety group, participants needed to be experiencing cancer-related anxiety, per self-report, as indicated by a score of 22 or higher on the PROMIS (Patient Reported Outcomes Measurement Information System) anxiety short form measure [32]. For the neuropathy group, participants needed to be experiencing CIPN, per self-report, as indicated by a score of 22 points or higher on the Functional Assessment of Cancer Therapy-Cognitive subscale (FACT-GOG-Ntx) CIPN items [33]. The study was powered to examine differences between groups with 200 people in each group (200 total for anxiety, 200 total for neuropathy).

#### Baseline and Follow-Up Assessments

**Figure 1:**
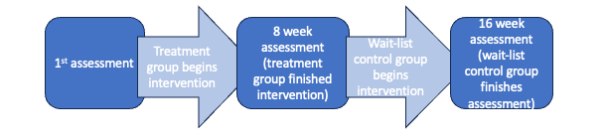
Timeline for baseline and follow-up data collection.

Figure 1 summarizes the overall timeline for baseline and follow-up data collection. For both the anxiety and neuropathy groups, data on demographics and medical history were collected at baseline. Baseline data collected included cancer status and treatment, past medical conditions, gender, ethnicity, and previous experience with mindfulness. For both the anxiety and neuropathy groups, all 3 surveys include questions about the quality of life (physical well-being, social or family well-being, emotional well-being, and functional well-being), anxiety (symptoms of anxiety and whether help was needed due to anxiety symptoms), fatigue (severity of fatigue and the impact of fatigue on daily functioning), pain (sensory, affective or emotional impact, and cognitive evaluation of pain), and mood (anxiety). The anxiety group received additional assessment questions: depression, mindfulness, and posttraumatic stress disorder (PTSD). The neuropathy group received questions about neuropathy: severity and impact of CIPN. Specific measures are listed below for each group.

#### Anxiety Group

Potential participants were screened with the PROMIS anxiety short form measure, an 8-item measure assessing anxiety on a Likert-type scale, with options ranging from “never” to “always” [32]. Pain is measured with the Pain Intensity Enjoyment of Life General Activity Scale, a 3-item measure assessing pain intensity, interference with general activity, and interference with enjoyment of life, with response options ranging from a 0-10 scale, with 0 being “no pain” or “does not interfere” and 10 being “pain as bad as you can imagine” or “completely interferes” [34]. Sleep-related impairment is measured using the PROMIS sleep-related impairment short form, an 8-item measure assessing sleep disturbance and sleep-related impairment [35]. Fatigue is measured with the brief fatigue inventory, a 9-item measure to rate fatigue on a 0-10 scale, with 0 being “no fatigue” or “does not interfere” and 10 being “as bad as you can imagine” or “completely interferes” [36]. Anxiety is measured with the General Anxiety Disorder 7-item measure of anxiety on a 5-point Likert-type scale [37]. Depression is measured with the Patient Health Questionnaire-9, a 9-item measure using 4 response options to assess depressive symptoms and behaviors [38]. PTSD is measured using the PTSD Checklist for DSM-5, a 20-item measure that assesses the 20 *Diagnostic and Statistical Manual of Mental Disorder* symptoms of PTSD [39]. Mindfulness is measured with the FFMQ, a 39-item measure of the 5 facets of mindfulness (observing, describing, acting with awareness, nonjudging of inner experience, and nonreactivity to inner experience), with answer options on a 1-5 scale ranging from “never or rarely true” to “very often or always true” [29]. For the follow-up assessments, the demographic questions are removed. A question regarding how much they have been practicing mindfulness (all groups), and a question about satisfaction with the intervention (for the intervention groups) are added.

#### Neuropathy Group

Potential participants were screened with the 11-item CIPN measure included in the FACT-GOG-Ntx [33]. The Functional Assessment of Cancer Therapy (FACT) scale is a well-validated quality of life measure with 27 items measuring physical, social or family, emotional, and functional well-being, with the FACT-GOC-Ntx scale including 11 additional items to measure the severity and impact of CIPN [33]. The 11-item FACT-GOG-Ntx data serves as both a screening tool and a baseline measure of CIPN. The rest of the FACT items (27 questions) are included in the baseline questionnaire, with the total measure being included at the 8- and 16-week assessment time points. At each assessment time point, participants are asked about any additional support services (such as support groups or clinical trials that they have been enrolled in, including anything they have done to treat CIPN. In order to assess anxiety, the 8-item PROMIS anxiety short form measure is used [32]. Fatigue is measured using the brief fatigue inventory [36]. The short-form McGill Pain Questionnaire (MPQ) is used as a brief measure of pain [40]. The original MPQ is a self-report questionnaire, consisting of 3 major classes of word descriptors of pain: sensory, affective or emotional impact, and cognitive evaluation of pain. The short-form MPQ was developed to provide an instrument that could be completed in less time than the MPQ but would still reflect both the sensory and affective dimensions of pain and has been shown to have high correlations with the original MPQ [40]. For the follow-up assessments, the demographic questions are removed. For the follow-up assessments, a question regarding how much they have been practicing mindfulness (all groups), and a question about satisfaction with the intervention (for the intervention groups) are added.

#### Intervention

Mindfulness Coach provides simple written instructions and a brief tutorial that introduces the major features, including a training plan, practice exercises, learning topics, and tracking via the app. Mindfulness exercises are delivered via brief, guided audio mindfulness exercises. This information has been arranged in such an order that it is helpful to have learned the material prior to any given session, although not necessary. The core meditation centers around the seated mindfulness practice, and as the sessions go on, users are asked to sit for longer periods of time, building physical and mental capabilities for meditation. Through this process, participants are instructed to bring nonjudgmental awareness to what they are experiencing in a basic sensorial way (eg, what they are feeling on their skin, hearing in the environment, and experiencing inside their body in regard to physical or emotional sensations or thoughts), with a primary aspect of this involving paying attention to and deepening one’s breathing. The app delivers 14 sessions (levels) that each include a prerecorded guided audio meditation exercise. The app also provides a mindfulness self-assessment tool through the FFMQ-SF [29], which users are reminded, but not required, to complete at levels 1, 7, and 14. Assessment feedback “quick tips” are provided based on the results of the FFMQ-SF.

**Figure 2:**
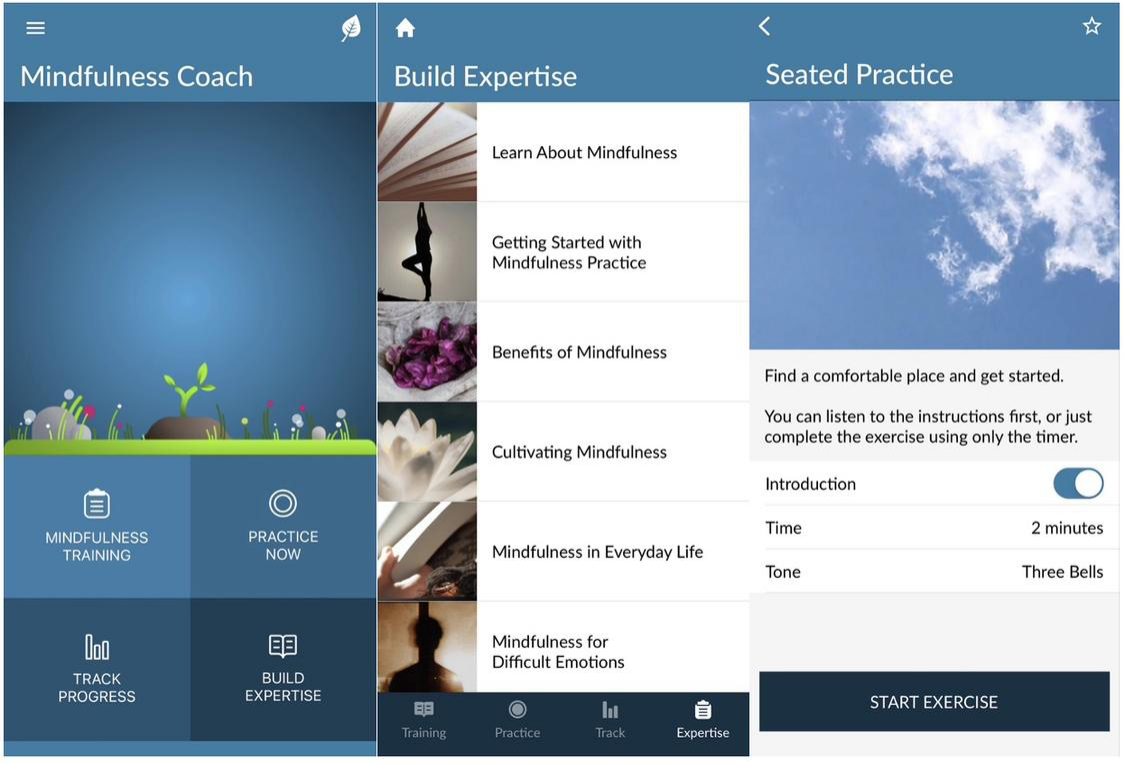
Screenshots of the Mindfulness Coach mobile app.

The intervention includes 8 weeks of Mindfulness Coach. (see Figure 2 for examples of features included in Mindfulness Coach). This practice is encouraged daily through the app and participants are asked to build the amount of time they are spending daily. There are many other audio recordings that are placed throughout the sessions, including meditations around scanning the body for sensations, awareness, and processing of difficult emotions, eating mindfully, and loving kindness. Participants are asked to use Mindfulness Coach for 8 weeks, either immediately (treatment group) or 8 weeks after the baseline assessment (waitlist control group).

### Ethical Considerations

The study was approved by the Western Internal Review Board (20131065). Informed consent was obtained from all participants included in the study. Study data were collected in Qualtrics and only relevant study personnel have access to the data. Participants receive a US $20 gift card after completing each of the 3 surveys, with a total incentive of US $60 for completing all 3 surveys. Reminder emails are sent to participants by a member of the study team.

## Results

### Overview

We will conduct the primary evaluation of the intervention, comparing changes in multiple outcomes between the treatment and waitlist control groups. Participants were randomized using a simple, open-label (nonblinded) randomization procedure. The intervention was individually guided and conducted electronically. We will follow an intent-to-treat approach, in which individuals are analyzed based on their randomization groups, regardless of adherence. Adherence effects will be examined in a subsequent analysis.

To account for repeated measures, we will run a mixed (both between- and within-subjects variables) linear or logistic model for each outcome. The independent variables are the randomization group (treatment vs waitlist control), time (baseline, 8 weeks, or 16 weeks), and the group by time interaction. The interaction term tests the intervention effect. For all analyses, the assumptions of the models will be checked and remedial measures (eg, transformations and nonparametric tests) will be taken as needed. Logistic models will assess dichotomous outcomes and linear models will assess continuous outcomes. Potential confounders (adherence, age, sex, and race or ethnicity) will be included. Where possible we will run the analyses within subgroups defined by these covariates, providing information on whether the intervention was more effective in certain groups (moderator effects). If there is evidence of nonrandom missing data, such as by differential dropout between groups, we will apply multiple imputation procedures. SAS (version 9.4; SAS Institute Inc) will be used to perform all analyses.

Data collection began in 2019 and was completed in 2023. We had very differential rates of recruitment for the differing arms with reaching near the target for the anxiety arm yet not for the cancer related neuropathy arm. This, along with other outcomes of the study will be reported in a separate manuscript.

### Power

Assuming a Bonferroni corrected (for 10 outcome measures) 2-tailed type I error rate (α) equal to .005 (0.05/10), 80% power, and 200 participants per group (400 total), a standardized difference (Cohen *d* effect size) of 0.37 will be detectable. Cohen *d* is the standardized difference and can be converted to the original units by multiplying the SD of the original variable.

## Discussion

We hope that this study will result in findings that are useful to improving the well-being of cancer survivors experiencing anxiety- and cancer-related neuropathy. If the study shows improvement in symptoms of anxiety or neuropathy in cancer survivors, it will allow for greater accessibility to mindfulness meditation via Mindfulness Coach, which is free, publicly available, and easily accessible through a mobile app. This will allow patients with cancer to access guided mindfulness meditation exercises in the comfort of their own homes and on their own time. This can be helpful for patients who may be too ill, lack resources, or live in a rural area where accessing a face-to-face mindfulness meditation intervention would be difficult or impossible. Results of the study may also be useful for clinicians working alongside patients with cancer or survivors searching for evidence-based, nonpharmacologic interventions to improve anxiety or neuropathy, with mindfulness meditation used alone or along with pharmacologic interventions.

Nonpharmacologic interventions, including exercise, yoga, and mindfulness meditation, have been studied for the treatment of psychological disorders, including anxiety, as a monotherapy or adjunctive therapy [41]. Current evidence supports the use of mindfulness meditation as an adjunctive therapy for people with an anxiety disorder, as there is limited evidence for mindfulness being used as a monotherapy for anxiety [41]. The general health benefits and low risk for harm justify the use of mindfulness as adjunctive therapy for patients with anxiety disorders and other mental health conditions such as depression [41]. At the current time, pharmacologic interventions for neuropathy are not highly effective and are not well tolerated by many patients due to various side effects [42,43]. First-line drugs used to treat neuropathy include antidepressants and anticonvulsants acting at voltage-gated calcium channels, which can have side effects including risk for cardiotoxicity, orthostatic hypotension, dry mouth, nausea, and confusion, among others [43]. In a randomized controlled trial involving 402 patients with neuropathy, 4 drugs commonly used for neuropathy of different drug classes (pregabalin, duloxetine, nortriptyline, and mexiletine) were tested for tolerance and treatment of neuropathic pain. The results of the study found that none of the drugs tested was clearly superior in performance or highly effective for treating neuropathic pain [42]. If results from this study show potential for mindfulness to be used as an alternative intervention to treat neuropathy, a larger number of people, including cancer survivors affected by CIPN, can access effective treatment without the side effects associated with medications commonly used to treat neuropathy.

Mobile apps have large dissemination potential and identifying apps that are found to be related to outcomes of interest is important given the ubiquitous nature of apps. Without identifying apps that are effective, apps that are available yet not effective can easily be reached. Findings from the currently described study will be published as soon as available.

## Abbreviations

CIPN: chemotherapy-induced peripheral neuropathy
FACT: Functional Assessment of Cancer Therapy
FACT-GOG-Ntx: Functional Assessment of Cancer Therapy-Cognitive subscale
FFMQ-SF: Five-Facet Mindfulness Questionnaire Short Form
MBSR: mindfulness-based stress reduction
MPQ: McGill Pain Questionnaire
PROMIS: Patient Reported Outcomes Measurement Information System
PTSD: posttraumatic stress disorder
UH: University of Hawai’i

## Data Availability

The data sets generated or analyzed during this study are not publicly available because it is ongoing. Findings from the currently described study will be published as soon as available.
